# Supplementary material for: Nomogram to Predict the Overall Survival of Colorectal Cancer Patients: A Multicenter National Study
Source: Int J Environ Res Public Health. 2021 Jul 21;18(15):7734. doi: 10.3390/ijerph18157734 (PMC8345484; doi:10.3390/ijerph18157734)
Supplement: Supplementary file 1 [file ijerph-18-07734-s001.zip › ijerph-1206037-supplementary.pdf]

Checklist item:

Section/topic	Item	Development or validation?	Checklist item
Title	1	D,V	This is a developing and validating a multivariable prediction model.<br>Target population are CRC patients who referred to three hospitals in Iran.<br>The two datasets are considered in training set and another dataset is regarded in testing set.
Abstract	2	D,V	The historical cohort study aimed to evaluate the survival predictors for all CRC patients using the CPH and established a novel nomogram to predict the Overall Survival (OS) of the CRC patients. Performance was assessed by the C-index (0.692 and 0.627 for train and test dataset). Also, Nomogram could assist clinical decision-making and prognosis predictions in patients with CRC.
Introduction			
Background and objectives	3a	D,V	According to GLOBOCAN 2020 data, the CRC can be regarded as the fourth most commonly diagnosed cancer globally.<br>Nomogram is a simple graphical representation of a statistical prediction model that generates a numerical probability of a clinical event and has been recently applied in prognosis-associated clinical studies with comparable results.<br>Several studies have implemented survival analysis, including frailty, time-varying Cox, and Cure models in CRC<br>This study was the first viewpoint on the predictive and prognostic factors regarding to nomogram presentation and OS for CRC in Iran to the best of our knowledge. Also, this is the first Iranian multicenter study that surveys both demographic and clinical traits of patients with CRC patients. The large sample size (n=1868) confirms a vast range of relationships with sufficient statistical analysis power in both train and test sets.
	3b	D,V	The goal of the historical cohort study, with such a large sample size population, was to apply Cox regression to assess the influence of crucial factors on CRC patients' survival rate who registered at three tertiary referral centers in Iran between 2006 and 2019. Then, the Nomogram diagram was drawn to generate the probability of survival in CRC patients.
Methods			
Source of data	4a	D,V	In this historical cohort study, we gathered both demographic information and clinical characteristics of 1868 patients who were diagnosed with CRC and referred to three tertiary Hospitals of Iran, Shahid Faghihi Hospital in Shiraz, Taleghani Hospital in Tehran as train sets and Imam Khomeini Hospital in Mazandaran as test set. Two first datasets were used to training set and another set was performed in testing set.

	4b	D,V	The data includes CRC patients' survival rate who registered at three tertiary referral centers in Iran between 2006 and 2019
Participant	5a	D,V	A total of 1654 of CRC patients, including Shiraz and Tehran cities used as train set, were included in this historical cohort study. Also, another dataset from Mazandaran was applied as Test set (n=219).
	5b	D,V	Patients who had a history of colorectal surgery for any reason except colorectal cancer were excluded. This means, the study includes all CRC patients who refer to three tertiary colorectal referral centers of Iran from 2006 to 2019.
	5c	D,V	The data includes a registry dataset that some treatments such as Chemotherapy, radiotherapy, immunotherapy, surgery were done on patients depending on stage, grade and types of cancer
Outcome	6a	D,V	The response variable was the time (months) elapsed from the cancer diagnosis until death. Several important clinical factors were included in the model, such as tumor size, the number of involved lymph nodes, distant metastasis, histology, type of treatment, history of polyp and CRC, comorbidity colon diseases (inflammatory bowel disease and irritable bowel syndrome), Diabetes Mellitus, tumor stage, location of tumor, and demographic variables such as sex, age, education level, smoking and alcohol consumption status, marital status, BMI.
	6b	D,V	In this historical cohort study, we gathered both demographic information and clinical characteristics of 1868 patients who were diagnosed with CRC and referred to three tertiary Hospitals of Iran.
Predictors	7a	D,V	Several important clinical factors were included in the model, such as tumor size, the number of involved lymph nodes, distant metastasis, histology, type of treatment, history of polyp and CRC, comorbidity colon diseases (inflammatory bowel disease and irritable bowel syndrome), Diabetes Mellitus, tumor stage, location of tumor, and demographic variables such as sex, age, education level, smoking and alcohol consumption status, marital status, BMI. Also, there are some missing data among variables
	7b	D,V	The response variable was the time (months) elapsed from the cancer diagnosis until death. Several important clinical factors were included in the model, such as tumor size, the number of involved lymph nodes, distant metastasis, histology, type of treatment, history of polyp and CRC, comorbidity colon diseases (inflammatory bowel disease and irritable bowel syndrome), Diabetes Mellitus, tumor stage, location of tumor, and demographic variables such as sex, age, education level, smoking and alcohol consumption status, marital status, BMI. Also, there are some missing data among variables
Sample size	8	D,V	In this historical cohort multicenter study that is population-based registers, we tried to collect as much sample size as we could to develop and also validate the nomogram. Also, the information for validity has been added.
Missing data	9	D,V	The total number of registered data was 1868 (1649 in train dataset and 219 in test dataset), and there was missing related to some variables. Therefore, the sum of frequency may not be equal to the total number and maybe slightly less.

Statistical analysis methods	10a	D	By consulting a doctorate of medicine, some important predictions were gathered from data registry from 2006 to 2016
	10b	D	The participants' clinical features are represented by reporting the mean with SD for continuous measures and frequency with proportions for categorical ones. The univariable CPH model was implemented to evaluate the effect of some essential factors on CRC patients. Those variables which had P < 0.2 in the univariable analysis were candidates for the multivariable regression analysis. All patients with missing data were removed from the study. The result of the multivariable Cox model was presented as a nomogram. A nomogram is a visual depiction of statistical models, such as the Cox proportional hazards model for survival data, which involves several independent variables to predict the survival probability.<br>The significance level for the statistical analysis was considered 0.05. The R 2.14.1 software (http://www.r-project.org) with the survival and rms packages was applied for statistical analysis. Also, the DynNom package was used to construct the dynamic nomogram
	10c	V	In the historical cohort study, with such a large sample size population, who registered at three tertiary referral centers in Iran between 2006 and 2019
	10d	D,V	The participants' clinical features are represented by reporting the mean with SD for continuous measures and frequency with proportions for categorical ones. The univariable CPH model was implemented to evaluate the effect of some essential factors on CRC patients. Those variables which had P < 0.2 in the univariable analysis were candidates for the multivariable regression analysis. All patients with missing data were removed from the study. The result of the multivariable Cox model was presented as a nomogram. A nomogram is a visual depiction of statistical models, such as the Cox proportional hazards model for survival data, which involves several independent variables to predict the survival probability.<br>The significance level for the statistical analysis was considered 0.05. The R 4.1.0 software (http://www.r-project.org) with the survival and rms packages was applied for statistical analysis. Also, the DynNom package was used to construct the dynamic nomogram.
	10e	V	The participants' clinical features are represented by reporting the mean with SD for continuous measures and frequency with proportions for categorical ones. The univariable CPH model was implemented to evaluate the effect of some essential factors on CRC patients. Those variables which had P < 0.2 in the univariable analysis were candidates for the multivariable regression analysis. All patients with missing data were removed from the study. The result of the multivariable Cox model was presented as a nomogram. A nomogram is a visual depiction of statistical models, such as the Cox proportional hazards model for survival data, which involves several independent variables to predict the survival probability.<br>The significance level for the statistical analysis was considered 0.05. The R 4.1.0 software (http://www.r-project.org) with the survival and rms packages

			was applied for statistical analysis. Also, the DynNom package was used to construct the dynamic nomogram.
Risk groups	11	D,V	We do not have any risk groups in our registered data.
Development vs. validation	12	V	Patients who had a history of colorectal surgery for any reason except colorectal cancer were excluded.
Results			
Participant	13a	D,V	Patients who had a history of colorectal surgery for any reason except colorectal cancer were excluded.<br>The number of patients diagnosed with CRC cancer between 2006-2019 (n=1937)<br>Excluded (n=69)<br>No history of CRC (n=29)<br>history of colorectal surgery for any reason except colorectal cancer (n=40)<br>Patients diagnosed with CRC (n=1868)<br>Training dataset (n=1649)<br>Testing dataset (n=219)
	13b	D,V	A total of 1649 of CRC patients, including Shiraz and Tehran cities used as train set, were included in this historical cohort study. Also, another dataset from Mazandaran was applied as Test set (n=219). Overall, 59.7% (n = 988) were male and 40.3% (n = 666) were female. The median follow-up time is 21.86 months (IQR: 9-37.2 and range 1, 179 months). The mean age of patients was 54 (14) years
	13c	V	In this regard, factors associated with survival are listed in Table 1 based on the univariable Cox regression. The table revealed that age, BMI, family history, tumor grade, stage of tumor, primary site, Diabetes history, T stage, N stage and types of treatment are significant in the univariable Cox model
Model Development	14a	D	A total of 1649 of CRC patients, including Shiraz and Tehran cities used as train set, were included in this historical cohort study. Also, another dataset from Mazandaran was applied as Test set (n=219). Overall, 59.7% (n = 988) were male and 40.3% (n = 666) were female. The median follow-up time is 21.86 months (IQR: 9-37.2 and range 1, 179 months). The mean age of patients was 54 (14) years
	14b	D	In this regard, factors associated with survival are listed in Table 1 based on the univariable Cox regression. The table revealed that age, BMI, family history, tumor grade, stage of tumor, primary site, Diabetes history, T stage, N stage and types of treatment are significant in the univariable Cox model
Model specification	15a	D	Present the full prediction model to allow predictions for individuals (i.e., all regression coefficients, and model intercept or baseline survival at a given time point)
	15b	D	The risk factors associated with survival are listed in Table 1 based on the univariable Cox regression. The table revealed that age, BMI, family history, tumor grade, stage of tumor, primary site, Diabetes history, T stage, N stage and types of treatment are significant in the univariable Cox model

Model performance	16	D	The multivariable Cox model's output presented that BMI, family history, grade tumor and stage of tumor are statistically significant (P < 0.05).<br>The HR of death for patients with BMI < 18 (underweight) is 94% more than those with overweight persons, which was significant (HR = 1.94, P <0.05). Also, the HR in normal weight person is 42% more than overweight person (HR = 1.42, P < 0.05). The HR in patients who do not have family history of cancer is 42% less than those who do not have family history (HR=.58, P=0.002).<br>A HR of tumor grade categories indicated that both moderate and poor differentiations had worse prognoses than poorly differentiated (HR = 1.5; HR = 2.67, P < 0.05).<br>By worsening the stage of tumor, the HR increase significantly in CRC patients. That means, the higher the stage of tumor, the higher the HR. The HR in patients with stage IV of CRC is about 3.2 times more than stage I of patients (HR=3.24, P=0.005)<br>Based on the results of multivariable analysis, we established a dynamic web-based nomogram to calculate the survival probability (Dynamic Nomogram (shinyapps.io), https://nbshiny.shinyapps.io/DynNomColorectal/). Using it, one can predict the long-term survival of patients with CRC (Figure 2). This statistic tool that combines all prognostic indexes represents a graphical model that simply calculates the individualized overall survival probability for CRC patients.
Model updating	17	V	The C-index for the nomogram, was calculated for train and test datasets. The C-index in train dataset was 0.692 (95% CI, 0.650–0.734). The demographic and clinical characteristics of all the CRC patients of test dataset, according to survival status were summarized in Table 3. Also, the C-index of testing set was estimated as 0.627(0.670, 0.686) which showed the nomogram provided good discernment.
**Discussion**			
Limitations	18	D,V	The limitation of the study is that some indispensable factors such as CEA level, Albumin, and Fibrinogen levels have not been recorded in the patients 'questionnaire.
Interpretation	19a	D	We tried to change the format of Nomogram to be user-friendly. For this purpose, we used the dynamic nomogram, presented in Figure 1. The relevant nomogram chart is available at the following web address. In this chart format, you can select the patient profile, then determine the year of survival, and selecting the prediction option, the probability of survival will be calculated and appeared in the predicted survival window. An image of dynamic Nomogram figure has also been added to the manuscript.<br>we established a dynamic web-based nomogram to calculate the survival probability (Dynamic Nomogram (shinyapps.io), https://nbshiny.shinyapps.io/DynNomColorectal/).
	19b	D,V	In the present study, the univariable and multivariable Cox regression models were applied, and then the nomogram diagram was constructed to predict OS,

			which was able to provide individualized estimates of potential survival benefit. The significant factors of the study are the BMI, family history of cancer, histology, depth of invasion. The C-index of train and test dataset was estimated 0.692 and 0.627 respectively. Also, time-dependent AUC was evaluated in separate times.
Implications	20	D,V	The limitation of the study is that some indispensable factors such as CEA level, Albumin, and Fibrinogen levels have not been recorded in the patients 'questionnaire.
Other information			
Supplementary information	21	D,V	TRIPOD (Transparent Reporting of a multivariable prediction model for Individual Prognosis Or Diagnosis) Statement
Funding	22	D,V	This research received no external funding

Items relevant only to the development of a prediction model are denoted by D, items relating solely to a validation of a prediction model are denoted by V, and items relating to both are denoted D;V.
